# Clinical value of perioperative myocardial strain assessment by transesophageal speckle-tracking echocardiography in patients undergoing off-pump coronary artery bypass grafting

**DOI:** 10.3389/fcvm.2026.1762935

**Published:** 2026-07-02

**Authors:** Hongbin Jiang, Yujie Shao, Xiaohan Zhang, Yi Cui, Weizhong Pan

**Affiliations:** 1 Shandong Second Medical University, Weifang, Shandong, China; 2 Hangzhou International Innovation Institute, Beihang University, Hangzhou, Zhejiang, China; 3 Department of Anesthesiology, Yantai Yuhuangding Hospital, Yantai, Shandong, China

**Keywords:** coronary artery disease, off-pump coronary artery bypass grafting, transesophageal echocardiography, transit-time flow measurement, two-dimensional speckle tracking imaging

## Abstract

**Objective:**

To evaluate the clinical value of perioperative global longitudinal strain (GLS) derived from transesophageal two-dimensional speckle-tracking echocardiography (2D-STI) in predicting biomarker-defined postoperative myocardial injury and early cardiac functional alterations in patients undergoing off-pump coronary artery bypass grafting (OPCABG).

**Methods:**

A total of 113 patients scheduled for OPCABG between February 2025 and February 2026 were enrolled. GLS was measured after anesthesia induction (T0), after graft reperfusion and protamine neutralization (T1), and 1.5 h after reperfusion (T2). Δ1 and Δ2 were calculated as T1−T0 and T2−T0. Transit-time flow measurement (TTFM) parameters were recorded after reperfusion. CK-MB, hs-cTnI, BNP, and left ventricular ejection fraction (LVEF) were assessed perioperatively. Patients were classified into high-risk and low-risk groups according to postoperative hs-cTnI thresholds for biomarker-defined myocardial injury. Spearman correlation, repeated-measures ANOVA, multivariable logistic regression, and receiver operating characteristic (ROC) analyses were performed.

**Results:**

Of the 113 patients, 47 were classified as high risk and 66 as low risk. Compared with the low-risk group, the high-risk group had significantly lower GLS values at T0, T1, and T2, as well as lower Δ1 and Δ2 values. Among TTFM parameters, only total PI differed significantly between groups. Repeated-measures ANOVA showed significant time, group, and interaction effects for GLS. GLS at all time points was negatively correlated with 48-hour peak CK-MB, hs-cTnI, and BNP, and was also associated with ICU length of stay. In multivariable logistic regression, GLS at T0, T1, and T2 remained independently associated with biomarker-defined postoperative myocardial injury, with odds ratios of 0.319, 0.437, and 0.408, respectively. Both Δ1 and Δ2 also showed independent predictive value, with Δ2 showing a stronger effect. ROC analysis showed that T0 GLS, T2 GLS, and Δ2 had predictive value for biomarker-defined postoperative myocardial injury, with AUCs of 0.885, 0.884, and 0.840, respectively.

**Conclusion:**

Perioperative GLS derived from transesophageal 2D-STI, particularly GLS after anesthesia induction, GLS at 1.5 h after graft reperfusion, and their dynamic changes, was associated with hs-cTnI elevation-defined postoperative myocardial injury and early cardiac functional alterations in patients undergoing OPCABG. Compared with TTFM, GLS provides complementary information on myocardial function and may help support intraoperative risk assessment.

## Introduction

Off-pump coronary artery bypass grafting (OPCABG) has been widely used in patients with coronary artery disease (CAD) because of its minimally invasive nature and its advantages in reducing postoperative complications, mortality, and medical costs (1–3). However, postoperative myocardial injury remains a significant challenge following OPCABG (4, 5). In existing approaches for the evaluation of myocardial injury and prognosis, conventional clinical indicators, electrocardiographic changes, and biomarkers provide useful information to some extent, but they often fail to comprehensively reflect microstructural and functional changes in the myocardium, thereby limiting their value in the early prediction of myocardial injury (6, 7).

Two-dimensional speckle tracking imaging (2D-STI) is a relatively new echocardiographic technique that tracks the spatial movement of acoustic speckles within the myocardium, enabling real-time quantitative assessment of myocardial deformation and motion to evaluate cardiac function (8). Recent studies have demonstrated that 2D-STI parameters have good predictive value for myocardial injury after coronary revascularization (9–11). In patients with CAD, 2D-STI has shown promising performance in preoperative assessment of cardiac function and in predicting long-term postoperative outcomes. Compared with conventional methods, 2D-STI allows more accurate evaluation of regional and global myocardial functional changes, thereby facilitating timely detection of myocardial injury and optimization of treatment strategies (12). Among these parameters, global longitudinal strain (GLS) is the most widely used and is highly sensitive for reflecting myocardial systolic and diastolic function (13). Although previous studies have explored the application of 2D-STI in CABG, studies evaluating short-term prognosis in OPCABG using transesophageal echocardiography remain limited. Conventional transthoracic echocardiography is often restricted by suboptimal image quality, whereas transesophageal echocardiography provides higher-resolution images and enables more detailed assessment of myocardial function. By combining 2D-STI with transesophageal echocardiography, anesthesiologists may monitor dynamic changes in myocardial function in real time and provide supplementary information for subsequent perioperative clinical decision-making.

The anatomic complexity of coronary artery lesions is an important factor influencing patient selection and prognosis in CABG, including OPCABG. Therefore, this study aimed to investigate the predictive value of perioperative 2D-STI parameters for biomarker-defined postoperative myocardial injury in patients undergoing OPCABG, thereby addressing an important gap in the current literature. By refining perioperative myocardial functional assessment, GLS may provide complementary information for early risk stratification without replacing conventional intraoperative assessment tools.

## Materials and methods

### Study population

A total of 113 patients who underwent OPCABG at Yantai Yuhuangding Hospital between February 2025 and February 2026 were enrolled in this study. The protocol was reviewed and approved by the Ethics Committee of Yantai Yuhuangding Hospital (Approval No. 2025-145), and written informed consent was obtained from all participants.

The inclusion criteria were as follows: (1) eligible for off-pump coronary artery bypass grafting; (2) left ventricular ejection fraction (LVEF) >40%; (3) age ≥18 years; (4) American Society of Anesthesiologists (ASA) physical status class II–III; (5) educational level no lower than primary school, with the ability to communicate effectively; and (6) informed consent obtained from both the patient and family members.

The exclusion criteria were as follows: (1) severe cardiac conduction disorders or concomitant valvular heart disease requiring simultaneous valve replacement or repair; (2) major systemic diseases, including malignancy, severe dysfunction of the brain, liver, or kidneys, autoimmune diseases, advanced heart failure, or coagulopathy; (3) alcohol or drug abuse; (4) history of depression or anxiety; (5) requirement for intraoperative conversion to cardiopulmonary bypass, intra-aortic balloon pump (IABP), or extracorporeal membrane oxygenation (ECMO) support; and (6) death within 24 h after admission; and (7) refusal to participate by the patient or family members.

### Anesthesia protocol

All patients received standardized general anesthesia and perioperative management. Patients fasted for 6–8 h and abstained from liquids for 4 h before surgery. Upon entering the operating room, intravenous access was established, and routine monitoring, including noninvasive blood pressure (BP), pulse oximetry (SpO_2_), and electrocardiography (ECG), was initiated. Under local anesthesia, a radial artery catheter was inserted for continuous invasive arterial pressure monitoring. After anesthesia induction, central venous catheterization was performed, and a transesophageal echocardiography probe was inserted for real-time monitoring (Vivid E95 color ultrasound diagnostic system; GE Vingmed Ultrasound, equipped with a 6VT-D transesophageal probe). An autologous blood recovery system was also prepared. Anesthesia induction was achieved via intravenous administration of midazolam (0.04 mg/kg), sufentanil (0.3 μg/kg), etomidate (0.2–0.4 mg/kg), and rocuronium (0.6 mg/kg). Endotracheal intubation was performed 1.5 min after drug administration, followed by mechanical ventilation via an anesthesia machine. The ventilation parameters were set as follows: an oxygen concentration of 60%, a tidal volume (VT) of 6–8 mL/kg, a respiratory rate of 10–14 breaths/min, and end-tidal carbon dioxide (PetCO_2_) maintained at 30–40 mmHg. Anesthesia maintenance was performed via continuous intravenous infusion of propofol (4–10 mg/kg/h) and sufentanil (0.05–0.2 μg/kg/h) combined with inhalation of sevoflurane (1%–1.5%). Intermittent intravenous administration of rocuronium was used to maintain muscle relaxation. The depth of anesthesia was monitored via the bispectral index (BIS), which was maintained between 40 and 60. Cerebral oxygen saturation was continuously monitored and maintained within ±20% of the baseline values. Vasoactive drugs were administered as needed to stabilize hemodynamics. At the end of surgery, patients were transferred to the cardiothoracic intensive care unit (ICU) with endotracheal intubation in place. Postoperative analgesia was achieved via a patient-controlled intravenous analgesia (PCIA) pump containing butorphanol.

### 2D-STI

Transesophageal echocardiography (TEE) images were acquired by the same anesthesiologist at the following three time points: (1) after hemodynamic stabilization following anesthesia induction and before the start of surgery (T0); (2) after completion of graft reperfusion and protamine neutralization (T1); and (3) 1.5 h after graft reperfusion, before the patient left the operating room (T2). TEE examination was performed using a Vivid E95 color ultrasound diagnostic system (GE Vingmed Ultrasound) equipped with a 6VT-D transesophageal probe. Mid-esophageal four-chamber (ME 4C), mid-esophageal two-chamber (ME 2C), and mid-esophageal long-axis (ME LAX) views were obtained. The operator was an anesthesiologist certified in TEE who had received standardized training before the study. During image acquisition, probe depth, multiplane angle, and sector orientation were adjusted to display the long axis of the left ventricle and the apical structures as completely as possible, thereby avoiding left ventricular foreshortening. Gain, imaging depth, and frame rate were also optimized to obtain high-quality images with clear endocardial borders and complete segmental visualization, with an image frame rate of 50–70 FPS. All images were acquired under relatively stable hemodynamic conditions and in the absence of obvious interference from surgical manipulation, and 3–5 consecutive cardiac cycles were recorded for subsequent analysis (14). The acquired data were analyzed via automated function imaging (AFI) software to calculate the global longitudinal strain (GLS) values. If segmental data were missing because of suboptimal image quality or tracking failure, the region of interest was manually adjusted and the analysis was repeated (15). All analyses were performed independently by the same trained operator, who was blinded to the patients' subsequent outcomes. Cases with more than two missing regions after manual adjustment were excluded from the analysis cohort. Retrospective reanalysis of echocardiographic images from a subset of patients yielded an intraclass correlation coefficient (ICC) of 0.968, indicating good reproducibility of echocardiographic image analysis. In this study, GLS measurements were performed only on analyzable images. Only images that met the requirements for image quality and had acceptable speckle-tracking results were included in the final analysis, whereas images with poor quality or unreliable tracking were excluded. Because this study did not prospectively and systematically record the feasibility rate of GLS acquisition, the proportion of excluded cases, or the proportion of inadequately tracked segments, these indicators could not be quantitatively analyzed. In the present study, the absolute value of GLS was used for analysis. Unless contraindicated, intraoperative TEE was routinely performed in all enrolled patients as part of our institutional standard perioperative monitoring protocol for OPCABG. Its main indications included assessment of global and regional ventricular function, evaluation of intravascular volume status, and detection of newly developed regional wall motion abnormalities.

### Observation indicators

For each patient, fasting peripheral venous blood samples were collected preoperatively for laboratory assessment of creatine kinase-MB (CK-MB), high-sensitivity cardiac troponin I (hs-cTnI), and B-type natriuretic peptide (BNP). Bedside transthoracic echocardiography was performed by experienced sonographers to obtain cardiac images and measure preoperative echocardiographic parameters, including left ventricular ejection fraction (LVEF), E/A ratio, E/E′ ratio, cardiac output (CO), stroke volume (SV), and tricuspid annular plane systolic excursion (TAPSE). After completion of all graft anastomoses and restoration of blood flow, the surgeon measured graft flow via a transit-time flowmeter. The flow volume (Q) and pulsatility index (PI) of each graft were recorded. When the measured values were unsatisfactory (Q < 20 mL/min or PI > 5) (16), the surgeon rechecked the graft anastomosis, corrected any technical issues, and repeated the measurements until optimal parameters were achieved. Postoperatively, peripheral venous blood samples were collected upon transfer to the intensive care unit (ICU) and again at 24 and 48 h after surgery for reassessment of CK-MB, hs-cTnI, and BNP levels. One week after surgery, the LVEF was re-evaluated. The length of ICU stay was also recorded. All the above indicators were collected by personnel who were blinded to intraoperative GLS and other related parameters.

### Grouping

According to the hs-cTnI thresholds reported in the VISION Cardiac Surgery study as being significantly associated with 30-day mortality, patients were divided into a high-risk group (24 h hs-cTnI > 5,670 ng/L or 48 h hs-cTnI > 1,522 ng/L) and a low-risk group (24 h hs-cTnI < 5,670 ng/L and 48 h hs-cTnI < 1,522 ng/L) (17).

### Statistical analysis

All the statistical analyses were performed using SPSS version 26.0 (IBM Corp., Armonk, NY, USA). The normality of continuous variables was assessed using the Shapiro–Wilk test. Data with normal distribution are expressed as the mean ± standard deviation and were analyzed using the independent-samples *t* test. Data with non-normal distribution are expressed as the median (interquartile range) [M (Q1, Q3)] and were analyzed using the Mann–Whitney U test. Categorical variables are presented as counts and percentages (%) and were compared using the chi-square test. Before repeated-measures ANOVA of GLS, Mauchly's test of sphericity was performed, followed by pairwise comparisons. Spearman's correlation analysis was conducted to evaluate the associations between 2D-STI parameters, TTFM parameters, and postoperative cardiac biomarkers, and Bonferroni correction was applied to adjust for multiple testing in the correlation analyses. With risk group as the dependent variable, multivariable logistic regression models were constructed. GLS at each time point and the changes in GLS between time points were entered into the models, with adjustment for covariates that differed significantly between groups (*P* < 0.05), including number of grafts, total PI, triglycerides, and preoperative LVEF, as well as clinically relevant covariates such as history of diabetes. Before regression analysis, multicollinearity was assessed, with a variance inflation factor (VIF) < 5 and tolerance >0.1 used as criteria indicating no severe multicollinearity. Receiver operating characteristic (ROC) curves were constructed to assess the predictive performance of 2D-STI parameters for myocardial injury following OPCABG. The Youden index was calculated to determine the optimal cutoff value, and the area under the curve (AUC) and its 95% confidence interval were calculated. DeLong's test was further used for pairwise comparisons of AUCs to assess whether differences between ROC curves were statistically significant. *P* < 0.05 was considered statistically significant.

## Results

### Baseline characteristics of patients in the high-risk and low-risk groups

Table 1 summarizes the baseline characteristics of patients in the high-risk and low-risk groups. Comparison of baseline characteristics between the two groups showed no statistically significant differences in age, sex distribution, BMI, smoking history, alcohol consumption history, history of hypertension, diabetes, or hyperlipidemia (all *P* > 0.05). However, the number of grafts differed significantly between the two groups (*χ*² = 12.138, *P* < 0.001), with a significantly higher proportion of patients receiving ≥3 grafts in the high-risk group than in the low-risk group (68.1% vs. 34.8%). Preoperative LVEF also differed significantly between the two groups (Z = −2.680, *P* = 0.007), with a higher preoperative LVEF in the high-risk group than in the low-risk group [65.00% (58.00%, 68.00%) vs. 60.00% (54.75%, 65.00%)]. Among lipid-related indicators, triglyceride levels were significantly higher in the high-risk group than in the low-risk group [1.50 (1.11, 1.88) mmol/L vs. 1.22 (0.95, 1.61) mmol/L, Z = −2.100, *P* = 0.036]. No statistically significant differences were observed between the two groups in the remaining laboratory parameters, echocardiographic parameters, or blood pressure parameters, including preoperative hs-cTnI, systolic blood pressure, diastolic blood pressure, total cholesterol, high-density lipoprotein, low-density lipoprotein, E/A ratio, E/E′ ratio, CO, SV, and TAPSE (all *P* > 0.05).

**Table 1:** Baseline characteristics of patients in the high-risk and low-risk groups.

Baseline characteristic	High-risk group (*n* = 47)	Low-risk group (*n* = 66)	t/Z/*χ*²	*P*-value
Age (years)	69 (58, 72)	67 (59, 72)	−0.111	0.912
Male sex	32 (68.0)	45 (68.2)	<0.001	0.991
BMI (kg/m²)	26.22 (23.32, 29.09)	25.56 (23.58, 28.36)	−0.300	0.764
Smoking history	20 (42.5)	24 (36.3)	0.442	0.506
Alcohol consumption history	12 (25.5)	16 (24.2)	0.024	0.876
History of hypertension	36 (76.6)	42 (63.6)	2.575	0.276
History of diabetes mellitus	19 (40.4)	26 (39.4)	0.012	0.912
History of hyperlipidemia	5 (10.6)	5 (7.6)	0.319	0.572
Number of grafts			12.138	<0.001
≤2 grafts	15 (31.9)	43 (65.2)		
≥3 grafts	32 (68.1)	23 (34.8)		
Preoperative left ventricular ejection fraction (%)	65.00 (58.00, 68.00)	60.00 (54.75, 65.00)	−2.680	0.007
Preoperative hs-cTnI (ng/L)	13.50 (4.76, 55.89)	12.42 (5.30, 83.55)	−0.134	0.893
Systolic blood pressure (mmHg)	139.81 ± 18.09	143.32 ± 20.00	−0.956	0.341
Diastolic blood pressure (mmHg)	76.00 (70.00, 88.00)	80.00 (72.00, 89.00)	−1.335	0.182
Total cholesterol (mmol/L)	3.97 (3.03, 4.90)	4.08 (3.20, 4.99)	−0.996	0.319
Triglycerides (mmol/L)	1.50 (1.11, 1.88)	1.22 (0.95, 1.61)	−2.100	0.036
High-density lipoprotein (mmol/L)	1.07 (0.89, 1.24)	1.09 (0.99, 1.37)	−1.524	0.128
Low-density lipoprotein (mmol/L)	2.11 (1.48, 2.87)	2.38 (1.64, 2.95)	−0.652	0.514
E/A ratio	0.80 (0.67, 1.00)	0.80 (0.67, 0.92)	−0.131	0.896
SV	86.55 ± 23.77	79.94 ± 25.91	1.384	0.169
CO	6.00 (5.05, 6.75)	5.40 (4.30, 6.70)	−1.221	0.222
E/E′ ratio	12.59 ± 4.09	11.35 ± 3.95	1.625	0.110
TAPSE ≥16 mm	47 (100.0)	65 (98.5)	0.718	0.397

Data are presented as mean ± standard deviation, median (Q1, Q3), or *n* (%). BMI, body mass index; hs-cTnI, high-sensitivity cardiac troponin I; E/A, ratio of early to late transmitral flow velocity; E/E′, ratio of early transmitral flow velocity to early diastolic mitral annular velocity; SV, stroke volume; CO, cardiac output; TAPSE, tricuspid annular plane systolic excursion.

### Comparison of 2D-STI and TTFM parameters between the high-risk and low-risk groups

Table 2 summarizes the 2D-STI and TTFM parameters in the high-risk and low-risk groups. Significant differences were observed in all 2D-STI parameters between the two groups (all *P* < 0.001). Specifically, GLS measured after anesthesia induction (T0), after graft reperfusion and protamine neutralization (T1), and 1.5 h after reperfusion (T2), as well as Δ1 and Δ2, were all greater in the low-risk group than in the high-risk group. Among the TTFM parameters, only total pulsatility index (PI) differed significantly between the two groups (Z = −2.651, *P* = 0.008), with a lower PI value in the low-risk group than in the high-risk group [4.350 (3.075, 5.725) vs. 5.500 (3.700, 7.500)]. No statistically significant differences were found in the remaining TTFM parameters (all *P* > 0.05).

**Table 2:** Comparison of 2D-STI and TTFM parameters between the high-risk and low-risk groups.

Parameter	High-risk group (*n* = 47)	Low-risk group (*n* = 66)	t/Z	*P*-value
T0 GLS	6.761 ± 1.101	8.942 ± 1.457	−8.652	<0.001
T1 GLS	7.998 ± 1.471	10.570 ± 1.908	−7.742	<0.001
T2 GLS	6.400 (5.300, 7.300)	9.800 (8.575, 10.625)	−6.942	<0.001
Δ1	1.200 (0.900, 1.600)	1.700 (1.300, 2.025)	−3.903	<0.001
Δ2	−0.400 (−0.700, 0.000)	0.600 (0.400, 0.700)	−6.188	<0.001
Total Q	113.000 (79.000, 155.000)	89.500 (60.000, 164.750)	−1.285	0.199
Total PI	5.500 (3.700, 7.500)	4.350 (3.075, 5.725)	−2.651	0.008
Mean Q	42.666 (27.000, 68.666)	42.250 (29.875, 66.375)	−0.542	0.588
Mean PI	1.875 (1.500, 2.800)	1.883 (1.537, 2.262)	−0.551	0.582
LIMA–LAD flow	50.000 (30.000, 97.000)	41.500 (28.000, 79.500)	−0.760	0.447
LIMA–LAD PI	1.700 (1.400, 2.100)	1.800 (1.500, 2.400)	−1.325	0.185

Data are presented as mean ± standard deviation, median (Q1, Q3), or *n* (%). 2D-STI, two-dimensional speckle-tracking imaging; TTFM, transit-time flow measurement; GLS, global longitudinal strain; PI, pulsatility index; Q, graft flow volume; LIMA, left internal mammary artery; LAD, left anterior descending artery.

### Repeated-measures analysis of GLS at three time points

Table 3 presents the results of repeated-measures analysis of variance for the 2D-STI parameters. Repeated-measures ANOVA showed a statistically significant difference in the overall perioperative GLS level between the high-risk and low-risk groups (F = 77.240, *P* < 0.001). A significant overall time effect was also observed, indicating that GLS values changed significantly over the course of surgery, from after anesthesia induction to after completion of graft reperfusion and protamine neutralization, and then to 1.5 h after graft reperfusion (F = 235.072, *P* < 0.001). In addition, the group × time interaction was statistically significant (F = 14.517, *P* = 0.005), suggesting that the trajectories or patterns of GLS change over time differed between the high-risk and low-risk groups. GLS measured after anesthesia induction, after graft reperfusion, and 1.5 h after graft reperfusion all differed significantly between the two groups (all *P* < 0.001). Within the high-risk group, GLS improved significantly after graft reperfusion compared with the post-anesthesia value (*P* < 0.001), but declined significantly 1.5 h later to a level lower than the immediate post-reperfusion value (*P* < 0.001), and was also lower than the post-anesthesia baseline (*P* = 0.01). Within the low-risk group, GLS likewise improved significantly after graft reperfusion compared with baseline (*P* < 0.001). Although it decreased again 1.5 h after reperfusion (*P* < 0.001), it remained significantly better than the post-anesthesia baseline value (*P* < 0.001) (Table 4).

**Table 3:** Repeated-measures analysis of variance of GLS at three time points.

Group	T0 GLS (%)	T1 GLS (%)	T2 GLS (%)	F	*P*-value
High-risk group (*n* = 47)	6.761 ± 1.101	7.998 ± 1.471	6.398 ± 1.510	114.547	<0.001
Low-risk group (*n* = 66)	8.942 ± 1.457	10.570 ± 1.908	9.389 ± 1.852	181.856	<0.001
F	74.855	59.942	83.216		
*P*-value	<0.001	<0.001	<0.001		
Between-group effect	F = 77.240, *P* < 0.001				
Within-group effect	F = 235.072, *P* < 0.001				
Group × time interaction	F = 14.517, *P* = 0.005				

Data are presented as mean ± standard deviation. GLS, global longitudinal strain.

**Table 4:** Pairwise comparisons of 2D-STI parameters within each group.

Group	Group A	Group B	Mean difference (A−B)	Standard error	*P*-value
High-risk group (*n* = 47)	T0	T1	−1.237	0.200	<0.001
	T0	T2	0.363	0.238	0.01
	T1	T2	1.600	0.274	<0.001
Low-risk group (*n* = 66)	T0	T1	−1.627	0.087	<0.001
	T0	T2	−0.477	0.103	<0.001
	T1	T2	1.180	0.100	<0.001

2D-STI, two-dimensional speckle-tracking imaging.

### Correlation analysis of cardiac function–related parameters, TTFM parameters, and 2D-STI parameters

Table 5 summarizes the correlations between 2D-STI parameters and cardiac function–related indicators. As shown, GLS measured at T0, T1, and T2 demonstrated moderate negative correlations with postoperative myocardial injury biomarkers (48-hour peak CK-MB and hs-cTnI) and heart failure biomarkers (48-hour peak BNP) (all *P* < 0.01). Additionally, the GLS measured at T1 showed a weak positive correlation with the left ventricular ejection fraction (LVEF) one week after surgery (*P* < 0.05), whereas the GLS measured at T0 showed a very weak positive correlation with the postoperative one-week LVEF (*P* < 0.05). GLS values were also negatively correlated with ICU length of stay (all *P* < 0.05). Both Δ1 and Δ2 demonstrated mild-to-moderate negative correlations with 48-hour peak CK-MB, hs-cTnI, and BNP levels (all *P* < 0.01). Δ1 was weakly positively correlated with postoperative one-week LVEF (*P* < 0.01) and very weakly negatively correlated with ICU length of stay (*P* < 0.05). After Bonferroni correction, statistical significance was defined as *P* < 0.005. GLS at all three time points, Δ1, and Δ2 remained significantly correlated with postoperative myocardial injury biomarkers, including 48-hour peak CK-MB and hs-cTnI; GLS at all three time points and Δ2 also remained significantly correlated with the heart failure biomarker 48-hour peak BNP. The remaining correlations did not retain statistical significance after correction.

**Table 5:** Correlation analysis between 2D-STI parameters and cardiac function–related indicators.

Indicator	T0 GLS	T1 GLS	T2 GLS	Δ1	Δ2
48-hour peak CK-MB	−0.683**	−0.656**	−0.692**	−0.436**	−0.537**
48-hour peak hs-cTnI	−0.673**	−0.627**	−0.654**	−0.357**	−0.499**
48-hour peak BNP	−0.389**	−0.389**	−0.385**	−0.228*	−0.289**
LVEF at 1 week postoperatively	0.190*	0.225*	0.167	0.264**	0.115
ICU length of stay	−0.234*	−0.254**	−0.207*	−0.195*	−0.143

Values are Spearman correlation coefficients. Unadjusted *P* values are indicated as follows: **P < 0.01 and *P < 0.05. After Bonferroni correction, statistical significance was defined as *P* < 0.005. *N* = 113. 2D-STI, two-dimensional speckle-tracking imaging; GLS, global longitudinal strain; CK-MB, creatine kinase-MB; hs-cTnI, high-sensitivity cardiac troponin I; BNP, B-type natriuretic peptide; LVEF, left ventricular ejection fraction.

Among the TTFM parameters, Table 6 summarizes the correlations between TTFM parameters and cardiac function–related indicators. Total PI was mildly positively correlated with postoperative myocardial injury biomarkers (48-hour peak CK-MB and hs-cTnI) and the heart failure biomarker (48-hour peak BNP) (all *P* < 0.05), and was very weakly positively correlated with ICU length of stay (*P* < 0.05). Total Q was mildly positively correlated with 48-hour peak hs-cTnI and 48-hour peak BNP (*P* < 0.05). Mean PI was mildly positively correlated with ICU length of stay (*P* < 0.05). After Bonferroni correction, only the correlations between total PI and postoperative myocardial injury biomarkers, including 48-hour peak CK-MB and hs-cTnI, remained statistically significant.

**Table 6:** Correlation analysis between TTFM parameters and cardiac function–related indicators.

Indicator	Total Q	Total PI	Mean Q	Mean PI	LIMA–LAD flow	LIMA–LAD PI
48-hour peak CK-MB	0.162	0.284**	−0.013	0.115	0.055	−0.104
48-hour peak hs-cTnI	0.220*	0.286**	0.027	0.104	0.117	−0.105
48-hour peak BNP	0.226*	0.215*	0.075	0.083	0.075	0.024
LVEF at 1 week postoperatively	−0.089	−0.087	−0.011	−0.042	−0.077	0.097
ICU length of stay	0.063	0.208*	0.046	0.208*	0.055	0.133

Values are Spearman correlation coefficients. Unadjusted *P* values are indicated as follows: **P < 0.01 and *P < 0.05. After Bonferroni correction, statistical significance was defined as *P* < 0.005. *N* = 113. TTFM, transit-time flow measurement; Q, graft flow volume; PI, pulsatility index; LIMA, left internal mammary artery; LAD, left anterior descending artery; CK-MB, creatine kinase-MB; hs-cTnI, high-sensitivity cardiac troponin I; BNP, B-type natriuretic peptide; LVEF, left ventricular ejection fraction.

### Multivariable logistic regression analysis of GLS and postoperative myocardial injury

Table 7 presents the multivariable logistic regression analyses of GLS at different time points in relation to postoperative myocardial injury. In each model, the tolerance values of the independent variables ranged from 0.558 to 0.967, and the variance inflation factor (VIF) ranged from 1.035 to 1.791. None exceeded the commonly used threshold (VIF < 5), indicating no significant multicollinearity among the independent variables. The results showed that GLS measured after anesthesia induction (T0), after graft reperfusion and protamine neutralization (T1), and 1.5 h after reperfusion (T2) was independently associated with the study outcome in all three models. The odds ratio (OR) for GLS was 0.319 at T0 (95% CI: 0.204–0.499, *P* < 0.001), 0.437 at T1 (95% CI: 0.314–0.608, *P* < 0.001), and 0.408 at T2 (95% CI: 0.290–0.573, *P* < 0.001). These findings indicate that GLS at all three time points was an independent factor associated with the study outcome, and that a greater absolute GLS value was associated with a lower risk of the outcome. Preoperative LVEF was also statistically significant in the T0 and T1 models (T0: OR = 1.074, 95% CI: 1.002–1.152, *P* = 0.043; T1: OR = 1.075, 95% CI: 1.005–1.150, *P* = 0.036), but did not reach statistical significance in the T2 model (OR = 1.071, 95% CI: 0.996–1.152, *P* = 0.065). History of diabetes, preoperative triglycerides, total PI, and number of grafts did not show consistent statistical significance across the three models.

**Table 7:** Multivariable logistic regression analysis of GLS at different time points and biomarker-defined postoperative myocardial injury.

Variable	T0 GLS model OR (95% CI)	*P*-value	T1 GLS model OR (95% CI)	*P*-value	T2 GLS model OR (95% CI)	*P*-value
History of diabetes mellitus (yes vs. no)	1.453 (0.477, 4.427)	0.511	1.348 (0.461, 3.947)	0.586	1.882 (0.599, 5.912)	0.279
Preoperative triglycerides (per 1 mmol/L)	1.541 (0.595, 3.990)	0.373	1.757 (0.703, 4.392)	0.228	1.675 (0.616, 4.557)	0.313
Preoperative LVEF (per 1%)	1.074 (1.002, 1.152)	0.043	1.075 (1.005, 1.150)	0.036	1.071 (0.996, 1.152)	0.065
Total PI (per 1 unit)	1.126 (0.844, 1.502)	0.419	1.115 (0.848, 1.468)	0.435	1.181 (0.875, 1.594)	0.276
Number of grafts (≥3 vs. ≤2)	2.580 (0.651, 10.227)	0.177	3.409 (0.915, 12.693)	0.068	2.150 (0.548, 8.436)	0.273
GLS (per 1% absolute value)	0.319 (0.204, 0.499)	<0.001	0.437 (0.314, 0.608)	<0.001	0.408 (0.290, 0.573)	<0.001

OR, odds ratio; CI, confidence interval; GLS, global longitudinal strain; LVEF, left ventricular ejection fraction; PI, pulsatility index.

Table 8 presents the multivariable logistic regression analyses of dynamic changes in GLS and postoperative myocardial injury. Δ1 and Δ2 were further entered into separate multivariable models. In the Δ1 model, GLS remained independently associated with the study outcome (OR = 0.362, 95% CI: 0.185–0.709, *P* = 0.003). In addition, preoperative LVEF (OR = 1.068, 95% CI: 1.008–1.132, *P* = 0.025) and number of grafts ≥3 (OR = 4.330, 95% CI: 1.383–13.556, *P* = 0.012) were also retained in the model. In the Δ2 model, GLS was likewise independently associated with the study outcome (OR = 0.253, 95% CI: 0.120–0.531, *P* < 0.001), whereas preoperative LVEF (OR = 1.063, 95% CI: 0.999–1.131, *P* = 0.053) and number of grafts (OR = 2.708, 95% CI: 0.853–8.597, *P* = 0.091) did not reach statistical significance. These results indicate that GLS remained stably associated with the study outcome, regardless of whether GLS at T0, T1, and T2 or dynamic change indices relative to T0 were used in the analysis.

**Table 8:** Multivariable logistic regression analysis of dynamic changes in GLS and biomarker-defined postoperative myocardial injury.

Variable	Δ1 model OR (95% CI)	*P* value	Δ2 model OR (95% CI)	*P*-value
History of diabetes mellitus (yes vs. no)	1.248 (0.509, 3.061)	0.628	1.777 (0.683, 4.623)	0.239
Preoperative triglycerides (per 1 mmol/L)	1.547 (0.827, 2.893)	0.172	1.441 (0.718, 2.890)	0.304
Preoperative LVEF (per 1%)	1.068 (1.008, 1.132)	0.025	1.063 (0.999, 1.131)	0.053
Total PI (per 1 unit)	1.119 (0.894, 1.401)	0.325	1.165 (0.913, 1.485)	0.220
Number of grafts (≥3 vs. ≤2)	4.330 (1.383, 13.556)	0.012	2.708 (0.853, 8.597)	0.091
GLS (per 1% absolute value)	0.362 (0.185, 0.709)	0.003	0.253 (0.120, 0.531)	<0.001

OR, odds ratio; CI, confidence interval; GLS, global longitudinal strain; LVEF, left ventricular ejection fraction; PI, pulsatility index.

### Predictive value of 2D-STI parameters for myocardial injury

Table 9 presents the optimal GLS cutoff values for predicting myocardial injury as determined using the Youden index. Receiver operating characteristic (ROC) curve analysis showed that GLS measured after anesthesia induction (T0), after graft reperfusion and protamine neutralization (T1), and 1.5 h after reperfusion (T2), as well as the dynamic change indices, all had predictive value for myocardial injury. Specifically, the AUC for T0 GLS was 0.885 (95% CI: 0.819–0.950, *P* < 0.001), with an optimal cutoff value of 8.15%, a sensitivity of 0.936, and a specificity of 0.788. The AUC for T1 GLS was 0.861 (95% CI: 0.792–0.930, *P* < 0.001), with an optimal cutoff value of 9.65%, a sensitivity of 0.894, and a specificity of 0.733. The AUC for T2 GLS was 0.884 (95% CI: 0.823–0.945, *P* < 0.001), with an optimal cutoff value of 8.35%, a sensitivity of 0.915, and a specificity of 0.788.

**Table 9:** Optimal cutoff values for predicting myocardial injury determined by the Youden index.

Parameter	AUC	95% CI	*P*-value	Optimal cutoff (%)	Specificity	Sensitivity	Positive predictive value (%)	Negative predictive value (%)
T0 GLS	0.885	0.819–0.950	<0.001	8.15	0.788	0.936	75.9	94.5
T1 GLS	0.861	0.792–0.930	<0.001	9.65	0.733	0.894	70.5	90.7
T2 GLS	0.884	0.823–0.945	<0.001	8.35	0.788	0.915	75.5	92.9
Δ1	0.719	0.623–0.814	<0.001	1.60	0.591	0.809	58.5	81.3
Δ2	0.840	0.755–0.925	<0.001	0.05	0.894	0.787	84.1	85.5
Preoperative EF	0.648	0.539–0.757	0.007	62.5	0.697	0.617	59.2	71.9

The Youden index was calculated as sensitivity + specificity − 1. The optimal cutoff value corresponds to the value at which the Youden index reached its maximum. AUC, area under the curve; CI, confidence interval; GLS, global longitudinal strain; EF, ejection fraction.

**Figure 1:**
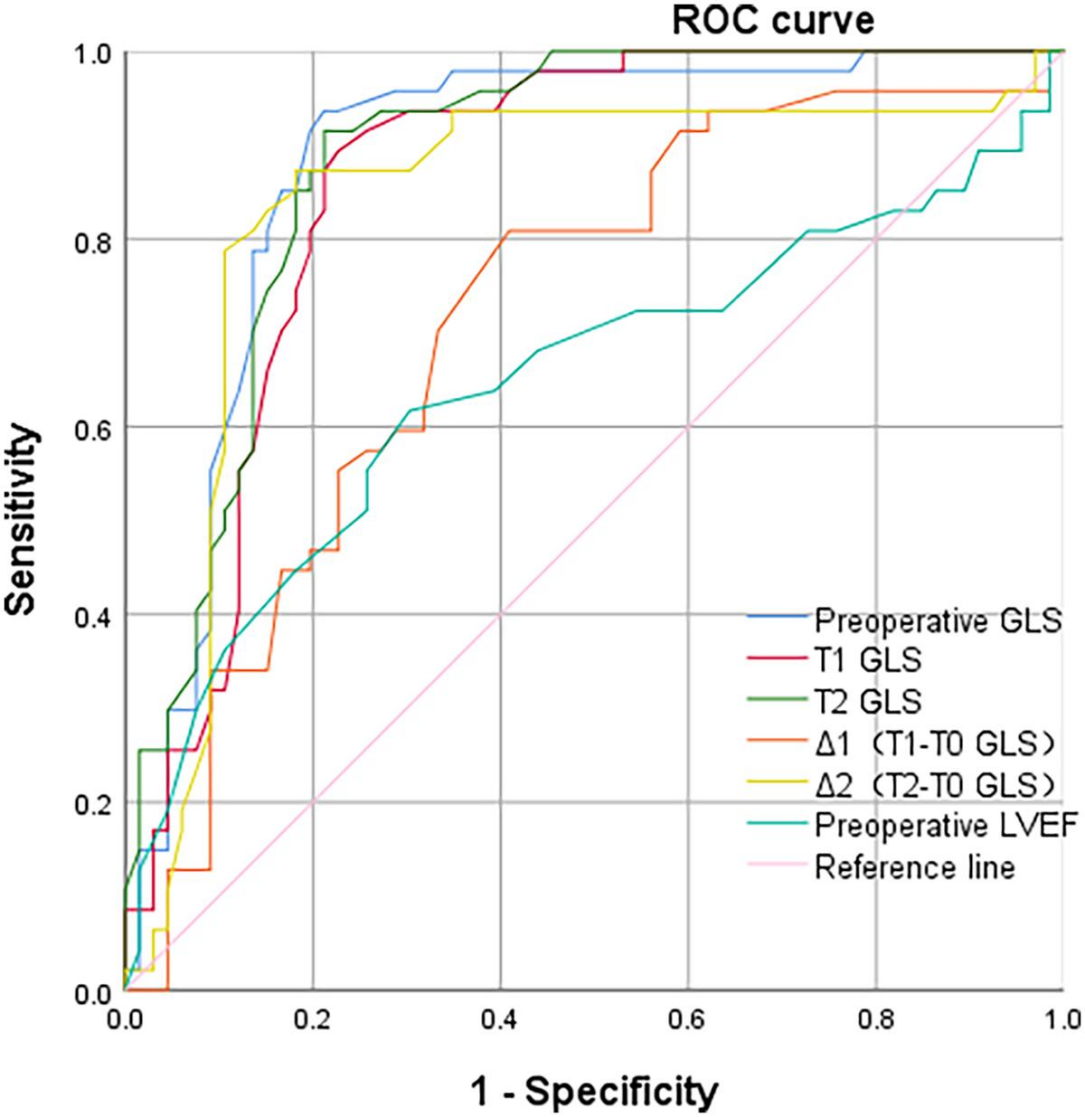
Predictive value of 2D-STI parameters for myocardial injury.

For the dynamic change indices, the AUC for Δ1 was 0.719 (95% CI: 0.623–0.814, *P* < 0.001), with an optimal cutoff value of 1.6, a sensitivity of 80.9%, and a specificity of 59.1%; the AUC for Δ2 was 0.840 (95% CI: 0.755–0.925, *P* < 0.001), with an optimal cutoff value of 0.05, a sensitivity of 78.7%, and a specificity of 89.4% (Figure 1). Further pairwise comparisons of AUCs (Table 10) showed that the AUC of Δ1 was significantly lower than those of preoperative GLS, post-reperfusion GLS, and GLS measured 1.5 h after reperfusion (all *P* < 0.05), and was also significantly lower than that of Δ2 (*P* = 0.005). In contrast, no statistically significant differences were observed among preoperative GLS, post-reperfusion GLS, GLS measured 1.5 h after reperfusion, and Δ2 (all *P* > 0.05).

**Table 10:** Pairwise comparisons of the areas under the ROC curves of GLS-related indicators.

Comparison	Difference in AUC	Standard error	*Z*	*P*-value	95% CI
T0 GLS vs. T1 GLS	0.024	0.258	1.583	0.114	−0.006 to 0.053
T0 GLS vs. T2 GLS	0.001	0.251	0.024	0.980	−0.038 to 0.039
T0 GLS vs. Δ1	0.166	0.286	3.177	0.001	0.064 to 0.268
T0 GLS vs. Δ2	0.044	0.277	0.911	0.363	−0.051 to 0.140
T1 GLS vs. T2 GLS	−0.023	0.254	−1.238	0.216	−0.060 to 0.014
T1 GLS vs. Δ1	0.142	0.287	3.338	0.001	0.059 to 0.226
T1 GLS vs. Δ2	0.021	0.279	0.480	0.632	−0.064 to 0.105
T2 GLS vs. Δ1	0.166	0.281	3.643	<0.001	0.076 to 0.255
T2 GLS vs. Δ2	0.044	0.271	1.297	0.195	−0.022 to 0.110
Δ1 vs. Δ2	−0.122	0.300	−2.830	0.005	−0.206 to −0.037

ROC, receiver operating characteristic; AUC, area under the curve; CI, confidence interval; GLS, global longitudinal strain.

## Discussion

CAD is caused by coronary artery stenosis or occlusion, which in turn leads to myocardial ischemia, and its incidence has been increasing gradually in China (18). It is well established that myocardial ischemia secondary to coronary artery stenosis results in regional wall motion abnormalities within the myocardial territory supplied by the affected coronary artery. Myocardial fiber shortening is reduced compared with that in healthy individuals, thereby leading to a lower absolute GLS value measured by 2D-STI (15). Ejection fraction (EF) is commonly used for preoperative and postoperative assessment of cardiac pumping function; however, it mainly reflects changes in ventricular ejection and is relatively insensitive to subtle functional impairment, resulting in incomplete evaluation in patients who may have heart failure with preserved ejection fraction (HFpEF). In contrast, 2D-STI, particularly GLS, can detect subtle changes in cardiac systolic function at an earlier stage and thereby predict postoperative outcomes, providing clinical evidence to support individualized postoperative management (19).

It should be noted that the primary endpoint used in this study was myocardial injury defined by postoperative high-sensitivity cardiac troponin I (hs-cTnI) thresholds, rather than clinically adjudicated perioperative myocardial infarction or other hard clinical endpoints. Therefore, the present findings more accurately reflect the ability of GLS to identify biomarker-defined postoperative myocardial injury. In recent discussions regarding the diagnostic criteria for myocardial injury/infarction after cardiac surgery, accumulating evidence has suggested that the association between troponin thresholds in traditional consensus definitions and patient prognosis urgently requires updating. In the setting of coronary artery bypass grafting (CABG), for example, the diagnosis of myocardial infarction requires the simultaneous presence of evidence of myocardial injury (such as troponin elevation) and evidence of myocardial ischemia, with troponin thresholds defined as >10 times the upper reference limit (URL) or ≥35 times the URL (with ischemic evidence) or ≥70 times the URL (isolated injury) (20, 21). However, these thresholds were established without clear prognostic justification and do not adequately account for differences in background myocardial trauma associated with different types of cardiac surgery. By contrast, the VISION Cardiac Surgery study, based on a multicenter prospective cohort, proposed that in patients undergoing isolated CABG or aortic valve replacement, the high-sensitivity cardiac troponin I (hs-cTnI) threshold associated with increased 30-day mortality was 220 times the URL on postoperative day 1 and 60 times the URL on postoperative day 2 or 3. Therefore, although the thresholds used in the present study are supported by relevant evidence, their clinical interpretation should remain cautious.

Under normal conditions, the absolute value of left ventricular GLS is typically greater than 18%–20%, with larger absolute values indicating stronger longitudinal myocardial shortening. In the present study, GLS was analyzed as a positive absolute value, with larger values representing better myocardial systolic function. Compared with previous studies, the absolute GLS values observed in this study were generally lower, suggesting impaired myocardial strain. This finding should be interpreted with caution. It may be speculated that the lower GLS values were related to the effects of general anesthetic agents and surgery after anesthesia induction, which may reduce myocardial contractility compared with the awake state and consequently lead to overall lower GLS values (22). Two mechanisms may account for this phenomenon. First, intravenous anesthetic agents such as propofol, together with positive-pressure ventilation, may reduce venous return and thereby decrease cardiac preload. This shortens the initial length of myocardial fibers and may subsequently increase the native strain value toward zero, corresponding to a lower absolute GLS value. Second, general anesthetic agents such as propofol may directly or indirectly depress myocardial contractility to varying degrees, thereby contributing to a reduction in the absolute value of GLS. However, the present study was not designed to further distinguish the independent effects of these factors. Therefore, the overall reduction in intraoperative GLS observed in this study is physiologically plausible, but interpretation of its absolute value should remain based on the specific perioperative physiological context. In addition, transesophageal echocardiography-derived GLS measured under general anesthesia cannot be directly compared with transthoracic echocardiography-derived GLS measured in the awake state.

Baseline analysis showed significant between-group differences in the following three indicators. Regarding the number of grafts, the proportion of patients receiving three or more grafts was significantly higher in the high-risk group than in the low-risk group (68.1% vs. 34.8%, *P* < 0.001). A greater number of grafts may reflect more diffuse coronary artery disease, long-term multiregional ischemia, impaired myocardial reserve, and greater procedural complexity, and may therefore be associated with a higher risk of postoperative myocardial injury. This is consistent with previous evidence identifying bypass graft number as a risk factor for postoperative myocardial injury or troponin release after CABG (23, 24). In the present study, preoperative LVEF was paradoxically higher in the high-risk group. This finding suggests that conventional LVEF may not fully reflect underlying subclinical myocardial dysfunction. Some patients may still have impaired longitudinal systolic function, microcirculatory dysfunction, and myocardial structural abnormalities despite preserved LVEF. In addition, ventricular remodeling and compensatory enhancement of contractility in the setting of chronic ischemia may also maintain EF at a relatively high level. On the other hand, this phenomenon may also be influenced by the limited sample size, selection bias, and limitations of conventional echocardiographic measurement. Therefore, a higher LVEF does not necessarily indicate better perioperative myocardial reserve (25); rather, this finding further suggests that GLS may provide additional value over LVEF in identifying occult myocardial functional abnormalities. Regarding triglycerides, their levels were higher in the high-risk group than in the low-risk group (1.50 vs. 1.22 mmol/L, *P* = 0.036). Hypertriglyceridemia may further impair coronary microcirculatory functional reserve by promoting endothelial dysfunction, increasing blood viscosity, and aggravating microcirculatory inflammatory responses, thereby making the myocardium more susceptible to ischemia-reperfusion injury.

In the present study, GLS measured after anesthesia induction, after graft reperfusion, and 1.5 h after reperfusion was moderately negatively correlated with the 48-hour peak CK-MB, hs-cTnI, and BNP levels (all *P* < 0.001), indicating that a lower absolute GLS value, namely poorer left ventricular myocardial strain function, was associated with a higher risk of postoperative myocardial injury and cardiac dysfunction. These findings are consistent with previous studies (10, 11, 26), supporting the role of GLS as a sensitive marker of subtle myocardial injury and early changes in cardiac function. In addition, GLS showed a weak or very weak positive correlation with LVEF at 1 week after surgery. This may be attributable to the limited sensitivity of LVEF as a global index of cardiac function, particularly in detecting subtle early functional changes. A possible explanation is that when cardiac function is impaired, both end-systolic and end-diastolic volumes may decrease to varying degrees; under such circumstances, EF may provide a misleading estimate of actual cardiac functional status.

Among the TTFM parameters, only PI differed significantly between the two groups (*P* = 0.008). However, in the multivariable logistic regression models, total PI did not reach statistical significance in any of the three time-point models. This finding is consistent with previous studies by Kim MS, Usta H, and others (27, 28), suggesting that although TTFM parameters can directly reflect graft patency, they have certain limitations in predicting postoperative myocardial injury and early outcomes. One possible explanation is that TTFM evaluates graft-level hemodynamics rather than the functional status of the myocardial cells themselves. Thus, even when graft flow and PI are within the normal range, distal target-vessel microcirculatory dysfunction or pre-existing impairment of hibernating myocardium may still lead to myocardial injury (29). In addition, TTFM measurements themselves may be subject to a certain degree of operator-related variability (30).

Repeated-measures ANOVA showed that the overall perioperative trajectory of GLS differed significantly between the high-risk and low-risk groups (group × time interaction, *P* < 0.05). Further within-group comparisons revealed that, although GLS in the high-risk group improved transiently after graft reperfusion, it returned to baseline 1.5 h later, whereas in the low-risk group it remained significantly better than baseline. These findings suggest that patients in the high-risk group had poorer myocardial functional reserve: although some recovery occurred after reperfusion, this improvement was difficult to sustain. By contrast, in the low-risk group, although cardiac function declined slightly after reperfusion, it remained overall improved relative to baseline.

This kinetic pattern of “transient improvement followed by decline” suggests that the initial functional benefit after reperfusion may not be sustained in high-risk patients. The subsequent decline in GLS may reflect limited myocardial reserve, impaired longitudinal shortening, and microcirculatory instability after reperfusion. Therefore, the perioperative trajectory of GLS may help identify patients with unstable reperfusion benefit and limited myocardial functional reserve.

The relationship between global longitudinal strain (GLS) at different perioperative time points and postoperative myocardial injury after OPCABG was further explored using multivariable logistic regression analysis. After adjustment for covariates including number of grafts, total PI, triglyceride level, and preoperative LVEF, GLS at T0, T1, and T2 showed a consistent direction of association. These findings indicate that a greater absolute GLS value was consistently associated with a lower risk of biomarker-defined postoperative myocardial injury. Among the dynamic change indices, Δ2 appeared to provide additional information beyond the acute improvement observed at T1. Overall, GLS may serve as a stable perioperative imaging marker for risk assessment after OPCABG. By comparison, the predictive performance of preoperative LVEF (AUC = 0.648) was markedly lower than that of GLS at any time point, further supporting that GLS may provide additional information beyond EF in the assessment of early myocardial functional impairment. On the other hand, conventional clinical risk factors such as history of diabetes, preoperative triglyceride level, and number of grafts did not show significant independent associations in the present study (*P* > 0.05), which may be related to sample size, study design, and interactions among variables.

ROC curve analysis showed that GLS measured at different perioperative time points, as well as its dynamic change indices, all had a certain discriminatory ability for biomarker-defined postoperative myocardial injury. These results suggest that perioperative GLS measured at different time points, particularly preoperative GLS, GLS measured 1.5 h after graft reperfusion, and Δ2, all have good predictive value; however, there is currently insufficient evidence to indicate that any single time-point parameter is clearly superior to the others. As a relatively stable observation time point after reperfusion, 1.5 h after graft reperfusion may help reflect intraoperative changes in myocardial function. At the same time, the dynamic difference between this value and preoperative GLS (Δ2) may provide additional information for risk assessment of biomarker-defined postoperative myocardial injury.

Despite the predictive value of GLS for postoperative outcomes demonstrated in the present study, several limitations should be acknowledged. First, this was a single-center exploratory observational study with a limited sample size. Therefore, potential overfitting, optimism bias, and selection bias may have led to overestimation of predictive performance in the ROC analysis. Second, the study endpoint was restricted to peak biomarker levels within 48 h after surgery, and no long-term follow-up data on recovery of cardiac function or major adverse cardiovascular events were available. Therefore, the prognostic value of GLS for long-term outcomes remains to be further clarified. Furthermore, the present study lacked both internal and external validation cohorts, and thus the stability and generalizability of the findings still require confirmation in larger prospective studies. In addition, this study did not systematically collect methodological data such as the feasibility rate of GLS acquisition, the proportion of excluded cases or inadequately tracked segments, nor did it quantitatively evaluate intraobserver or interobserver variability; therefore, the assessment of the feasibility and reproducibility of strain measurements remains insufficient. Future multicenter studies with larger sample sizes are warranted to further validate the predictive value of GLS and to incorporate additional imaging and biochemical markers into multivariable analyses.

## Conclusion

In summary, the dynamic changes in GLS derived from 2D-STI during the perioperative period of OPCABG showed predictive value for biomarker-defined postoperative myocardial injury and early cardiac functional alterations. Unlike TTFM, GLS provides myocardial functional information beyond graft flow parameters and may help support postoperative risk assessment and individualized management after OPCABG.

## Data Availability

The raw data supporting the conclusions of this article will be made available by the authors, without undue reservation.
